# Fluid flow in porous media using image-based modelling to parametrize Richards' equation

**DOI:** 10.1098/rspa.2017.0178

**Published:** 2017-11-22

**Authors:** L. J. Cooper, K. R. Daly, P. D. Hallett, M. Naveed, N. Koebernick, A. G. Bengough, T. S. George, T. Roose

**Affiliations:** 1 Bioengineering Sciences Research Group, Faculty of Engineering and the Environment, University of Southampton, Southampton, UK; 2 Institute of Biological and Environmental Sciences, University of Aberdeen, Aberdeen, UK; 3 The James Hutton Institute, Invergowrie, Dundee, UK; 4 School of Science and Engineering, University of Dundee, Dundee, UK

**Keywords:** image-based modelling, porous media, Richards' equation

## Abstract

The parameters in Richards' equation are usually calculated from experimentally measured values of the soil–water characteristic curve and saturated hydraulic conductivity. The complex pore structures that often occur in porous media complicate such parametrization due to hysteresis between wetting and drying and the effects of tortuosity. Rather than estimate the parameters in Richards' equation from these indirect measurements, image-based modelling is used to investigate the relationship between the pore structure and the parameters. A three-dimensional, X-ray computed tomography image stack of a soil sample with voxel resolution of 6 μm has been used to create a computational mesh. The Cahn–Hilliard–Stokes equations for two-fluid flow, in this case water and air, were applied to this mesh and solved using the finite-element method in COMSOL Multiphysics. The upscaled parameters in Richards' equation are then obtained via homogenization. The effect on the soil–water retention curve due to three different contact angles, 0°, 20° and 60°, was also investigated. The results show that the pore structure affects the properties of the flow on the large scale, and different contact angles can change the parameters for Richards' equation.

## 1. Introduction

Richards' equation is widely applied to model partially saturated fluid flow through porous media, such as soil. Parametrization of Richards' equation is challenging, primarily because of the difficulties in relating the parameters to easily measurable experimental values. The saturation form of Richards' equation for homogeneous soils [1] can be written in the form (from [2])

1.1 $$∥\Omega_{p}∥\frac{\partial S}{\partial t_{R}}-\nabla \cdot [D_{R}(S)\nabla S-k_{R}K_{R}(S){\hat{e\;e}}_{3}]=0,$$

where ∥*Ω*_p_∥ is the volume of the pore space per unit volume of soil, *S* is the relative water saturation of this pore space, *t*_R_ is the time, *D*_R_(*S*) is the soil moisture diffusivity, *k*_R_ is the relative permeability of the wetting fluid, *K*_R_(*S*) is the hydraulic conductivity and ${\hat{e\;e}}_{3}$ is the unit vector in the vertical direction, defined as positive in the downward direction, i.e. the direction of gravitational drainage. *D*_R_(*S*) and *K*_R_(*S*) are often parametrized using an experimentally measured soil–water characteristic curve (SWCC).

**Figure 1.**
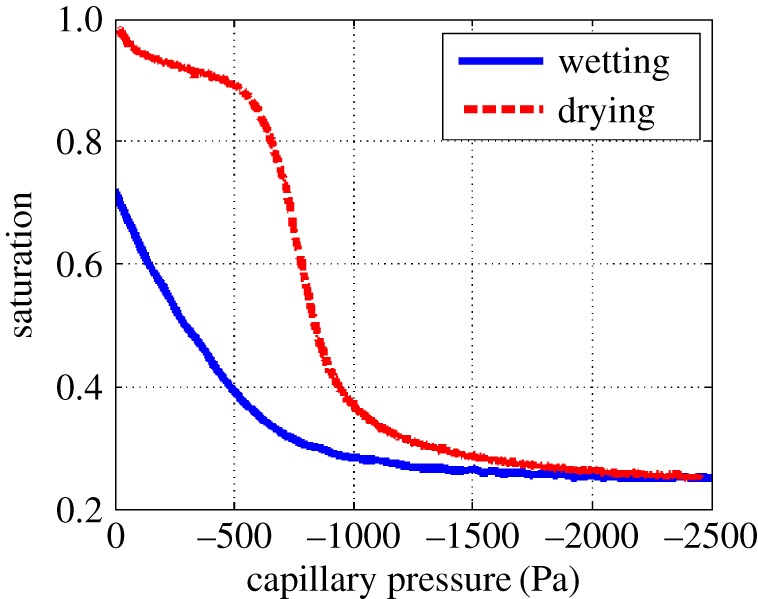
Experimentally measured soil–water characteristic curve for a sand-textured Eutric Cambisol collected from Abergwyngregyn, UK (53°14′ *N*, 4°01′ W). Details of the experimental procedure can be found in the electronic supplementary material. (Online version in colour.)

The SWCC relates the water content to the matric potential. The matric potential is the negative pressure that is applied to all water within a partially saturated porous media due to the surface tension at the air–water interfaces. The SWCC leads to an estimate of the pore structure; however, the experimentally measured values can vary spatially within a sample and often bulk measurements are made in order to capture the large-scale behaviour. The SWCC can be measured by applying a known pressure to a sample, allowing water to enter or leave the sample and then measuring the amount of moisture that remains in the sample. This results in several discrete points along the SWCC being measured and then interpolated by fitting models, such as Van Genuchten [3] or Brooks & Corey [4]. These models aim to replicate the intrinsic reverse ‘S’ shape of the SWCC (figure 1), i.e. for a drying curve, a plateau at high saturations corresponding to a small matric potential, a negative gradient as the water drains out of the soil, and a second plateau where a large matric potential is required to fully dry the soil [5]. Wetting curves have a similar shape to the drying curves, but hysteresis due to pore shape and connectivity offsets the two curves. In some cases, the models include two or three fitted parameters that are not independent. These fitted expressions are empirical and not directly related to a particular physical property of the porous media. The pore structure characterized with these approaches is therefore incompletely described and poorly parametrized, creating challenges to subsequent modelling of water retention and (even more so) to fluid flow. As well as the intrinsic shape of the SWCC, Haines' jumps are another behaviour that has been observed experimentally when wetting or drying porous media. Haines' jumps occur when a fluid phase moves abruptly from one configuration to another topologically different configuration due to the soil geometry, and associated with this is a drop in capillary pressure [6].

Richards' equation was originally derived phenomenologically by observing the behaviour of hydraulic conductivity and matric potential [1]. More recently, Daly & Roose [7] have shown that Richards' equation can be derived by coupling the Cahn–Hilliard phase field equation [8–10] with Stokes' equations and using homogenization [11]. Homogenization is a mathematical technique used to determine the macroscale properties of a problem described on the microscopic scale and has been used to model fluid flow in porous media [12,13]. This resulted in a form of Richards' equation that is dependent on the underlying geometry of the porous media, the contact angle between the air–water interface and the solid material, and the initial location of the interface. The advantage of this method over empirical fitting of data is that it allows for the investigation of the underlying causes of the behaviour observed at the macroscale by accounting for the microscale explicitly. The equations could be applied to any porous medium within a suitable range of parameters. The derivation of the equations relies on the following assumptions [7]:

— the interface between the two fluids has a finite thickness which is small compared with the geometry to which the model is applied;
— the interface position is determined largely by capillary forces;
— the mass of both fluids is constant;
— there is a no-slip condition on the surface of the soil particles, i.e. the fluid velocity on the soil particle surfaces is zero; this is not essential, a finite slip condition could be used instead;
— the initial position of the fluid–fluid interface is known;
— the contact angle is constant and known.

The contact angle is the only experimentally measured value present in the non-dimensional equations for calculating the position of the interface; therefore, it is only the contact angle and the geometry, i.e. the pore structure, that can affect the behaviour of the SWCC in this model. Although water flow and retention in soils often assumes that the contact angle is 0°, and is therefore considered not important, a large body of recent research has observed that contact angles between 0° and 90° are commonplace in soils [14,15].

The contact angle is a challenging value to measure as it can be affected by particle geometry, surface morphology and surface chemistry, and is also known to vary with hydration status [16]. For porous media flow, the influence of the contact angle was initially derived based on the notion of bundles of smooth capillary tubes. As early as the work of Philip [17] this was recognized as an ‘over-simplification’ of reality because of the impact of particle roughness. Since this early work, the impact of particle roughness on the soil–water contact angle has received considerable attention. This includes discussion of the difference between the small-scale (i.e. smooth) contact angle of a surface and the larger-scale contact angle when many interacting particles and their macroscopic surface topology influence roughness [14,15]. We therefore adopt two interpretations of the contact angle, which shall be called the theoretical and experimental contact angles. The theoretical contact angle is between the fluid–fluid interface and a single smooth soil particle surface, and it is this angle that is required in the model. The experimental contact angle is the angle that it is possible to measure in experiments with soils. Experimental approaches used to measure the contact angle in soil and other porous media are either direct measures of a water drop contact angle (sessile drop) or indirect measures from immersion tests of flat surfaces coated with groups of particles (Whilhemy plate) or the rate of liquid flow into a column (capillary rise) [18]. These provide different results due to differences in test geometry. Owing to the difficulties in measuring the contact angle at suitably high resolution, i.e. against an ideal smooth soil particle surface, it is often assumed that the theoretical contact angle is the same as the experimentally measured contact angle [14]. Here, to investigate the effect of different contact angles on the SWCC, three different contact angles, consistent with Czachor *et al.* [14] and McHale *et al.* [15], have been used to calculate the position of the water and air phases.

Daly & Roose [7] implemented the model on an idealized soil particle with contact angles of 70°, 90° and 110°. With high-resolution imaging, it would be feasible to obtain realistic soil geometries and combine these with computational modelling. In this way, different soil structures could be modelled to investigate how the geometry of the soil and contact angle impact the SWCC and the parameters for Richards' equation. This will allow for comparisons to be made between different soils that will improve our understanding of why they have different hydraulic properties. Imaging in combination with mathematical and numerical modelling has been used to investigate porous media. An example is a study by Tahmasebi *et al.* [19,20], where the three-dimensional (3D) morphology of a shale sample was derived from two-dimensional images and Stokes' equation was used to model fluid flow through the pores in order to estimate the effective permeability of the sample. Daly *et al.* [13] used three-dimensional X-ray computed tomography (3D XCT) images to estimate the hydraulic conductivity of soil samples, and Daly *et al.* [21] used 3D XCT images to calculate the effective diffusion and nutrient uptake by roots and root hairs. Further examples of combining imaging and modelling in porous media research have been extensively reviewed in Blunt *et al.* [22] and Roose *et al.* [23].

Here, a proof-of-concept study is presented where the equations from Daly & Roose [7] were applied to a computational mesh created from a 6 *μm* resolution 3D XCT image of a sand-textured soil sample. An advantage of this method is that it makes optimization of water movement possible, with respect to soil geometry and the contact angle between the fluid–fluid interface and solid soil particle surfaces. Determining which soil geometries or contact angles allow improved water uptake by plants makes it possible to select plants with root traits that can manipulate their environment to achieve these geometries, for example by root extension or root hairs, or contact angles, for example by the quantity of root exudate released. It also enables visualization of fluid movement within the soil geometry, leading to insights, particularly into hysteresis, in the underlying behaviour of the system.

## 2. Material and methods

### (a) Imaging

The imaging data used in this study have previously been published in Daly *et al.* [21], and hence only a brief description is presented here. The soil was a sand-textured Eutric Cambisol collected from Abergwyngregyn, UK (53°14′ N, 4°01′ W). The soil was sieved to less than 5 mm, autoclaved and air-dried at 23±1°*C* for 2 days and sieved to particle sizes between 1680 and 1000 μm [21]. The sieved soil was poured into a 6 mm diameter syringe barrel and given a few taps to make it settle in the barrel. No compaction was applied, resulting in a loose soil packing. This sample preparation results in a packed bed of soil aggregates, similar to a loose seedbed, consisting of a bimodal pore distribution of inter- and intra-aggregate pore space. The soil sample was imaged at the TOMCAT beamline at the Swiss Light Source with an image resolution of 1.2 μm and then downsampled to a resolution of 6 μm in order to remove noise and reduce computational cost. At this resolution only the inter-aggregate pore space was visualized and pores less than approximately 6 μm were not resolved. The soil and pore space were segmented using the trainable plug-in WEKA in Fiji [24]. Further details of the soil, imaging parameters and segmentation techniques can be found in Daly *et al.* [21]. The segmented geometry was used to create computational meshes using ScanIP 4.0 (Simpleware Ltd, UK), a commercial meshing software.

### (b) Model overview

**Figure 2.**
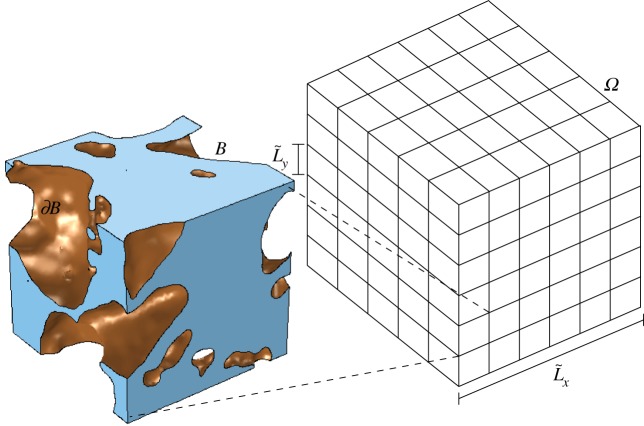
*Ω* is the whole porous domain with macroscopic length scale ${\tilde{L}}_{x}$. It is made up of regularly repeating units of volume ${\tilde{L}}_{y}^{3}$. *B* is the total fluid domain, i.e. air and water, of the unit, which is assumed to be connected; ∂*B* is the surface of solid soil particles, which is assumed to be smooth. (Online version in colour.)

The model developed by Daly & Roose [7] shows that, by homogenizing the Cahn–Hilliard–Stokes two-fluid equations, Richards' equation can be derived and parametrized by a series of cell problems that account for the soil structure. This is useful for solving problems where calculating the microscale equations on the full domain would be too computationally intensive to solve. Daly & Roose [7] assumed that the porous media domain, *Ω*, had a macroscale length of ${\tilde{L}}_{x}$ and was formed of regularly repeating microscale units with a length of ${\tilde{L}}_{y}$ such that ${\tilde{L}}_{y}/{\tilde{L}}_{x}=\epsilon \ll 1$. This is illustrated in figure 2. The method of homogenization assumes that these two length scales are independent of each other and results in a set of representative cell problems that can be solved on a single microscale unit. The macroscale properties that apply to *Ω* can then be determined.

Daly & Roose [7] derived a dimensionless form of Richards' equation. The equations are presented here in a rescaled form:

2.1a $$∥B∥\frac{\partial S}{\partial \tau_{1}}-\nabla \;\nabla_{x}\cdot \left[\frac{a(S)}{\mathrm{Ca}}K(S)\nabla \;\nabla_{x}S-b(S)\nabla \;\nabla_{x}p_{0}-b_{g}(S){\hat{e\;e}}_{3}g\right]=0,\;x\in \Omega ,$$

and

2.1b $$\;\nabla \;\nabla_{x}\cdot \left[\frac{a(S)}{\mathrm{Ca}}\bar{K}(S)\nabla \;\nabla_{x}S-\bar{b}(S)\nabla \;\nabla_{x}p_{0}-{\bar{b}}_{g}(S){\hat{e\;e}}_{3}g\right]=0,\;x\in \Omega ,$$

where ∥*B*∥ is the volume of the pore space in the microscale unit, *B* is the total fluid domain of the microscale unit, *τ*_1_ is the slow time scale caused by macroscopic applied pressure gradients, ∇_*x*_ is the gradient operator on the macroscale, *p*_0_ is the leading-order term for combined pressure and *g* is the scaled gravitational acceleration. Here, *p*_0_ is referred to as the combined pressure as it enforces the incompressibility of the fluid, both air and water together, and includes the external pressure applied to the system [7,25]. The capillary number is defined as

2.2 $$\mathrm{Ca}=\frac{{\tilde{L}}_{x}}{{\tilde{L}}_{y}}\frac{{\tilde{\eta }}^{\left(1\right)}[u\;u]}{\alpha \tilde{\gamma }},$$

where ${\tilde{\eta }}^{\left(1\right)}$ is the viscosity of fluid 1, $\alpha =6\sqrt{2}$, $\tilde{\gamma }$ is the surface tension and

2.3 $$[u\;u]=\frac{{\tilde{\rho }}^{\left(1\right)}\tilde{g}{\tilde{L}}_{y}^{2}}{4{\tilde{\eta }}^{\left(1\right)}},$$

is the velocity scaling, where ${\tilde{\rho }}^{\left(1\right)}$ is the density of fluid 1 and $\tilde{g}$ is gravity. The other functions are given by

2.4a $$\begin{array}{rl} a(S) & \;=-\frac{\delta \mu_{0}}{\delta S}, \end{array}$$

2.4b $$\begin{array}{rl} K(S) & \;=\int_{B}\phi_{0}\kappa \;\kappa_{k}^{\mu }⊗{\hat{e\;e}}_{k}\;dy\;y, \end{array}$$

2.4c $$\begin{array}{rl} b(S) & \;=\int_{B}\phi_{0}\kappa \;\kappa_{k}^{p}⊗{\hat{e\;e}}_{k}\;dy\;y, \end{array}$$

2.4d $$\begin{array}{rl} b_{g}(S) & \;=\int_{B}\phi_{0}\kappa \;\kappa^{g}⊗{\hat{e\;e}}_{3}\;dy\;y, \end{array}$$

2.4e $$\begin{array}{rl} \bar{K}(S) & \;=\int_{B}\kappa \;\kappa_{k}^{\mu }⊗{\hat{e\;e}}_{k}\;dy\;y, \end{array}$$

2.4f $$\begin{array}{rl} \bar{b}(S) & \;=\int_{B}\kappa \;\kappa_{k}^{p}⊗{\hat{e\;e}}_{k}\;dy\;y, \end{array}$$

2.4g $$\begin{array}{rl} \text{and}\;{\bar{b}}_{g}(S) & \;=\int_{B}\kappa \;\kappa^{g}⊗{\hat{e\;e}}_{3}\;dy\;y, \end{array}$$

where *μ*_0_ is the leading-order term for the capillary pressure and *δ*/*δS* is the functional derivative with respect to saturation. Here, $\kappa \;\kappa_{k}^{\mu }$, $\kappa \;\kappa_{k}^{p}$ and *κ*^*g*^ are the velocity coefficients of both the air and the water driven by capillary pressure, the combined pressure and gravity, respectively. By *k*=1,2,3 we define the direction in which the unit force is applied with respect to the major axes. These coefficients are the average velocities for a unit pressure drop. These terms are calculated from a series of cell problems, which refers to the set of equations solved on the periodic unit cell [11], i.e. the microscale domain. The cell problems derived for this set of equations are presented in Daly & Roose [7] and will be described in detail in §2c. By *ϕ*_0_ we denote the leading-order term of the phase field, where *ϕ*_0_=1 in fluid 1, e.g. water, and *ϕ*_0_=0 in fluid 0, e.g. air, and ⊗ is the tensor product. By *S* we denote the relative water saturation, defined as

2.5 $$S=\frac{1}{∥B∥}\int_{B}\phi_{0}\;dy\;y,$$

where d***y*** is a 3D element. Equation (2.4a) for *a*(*S*) describes the gradient of the SWCC. Equations (2.4b)–(2.4d) are the velocity coefficients for the water phase driven by capillary pressure, combined pressure and gravity, respectively. Equations (2.4e)–(2.4g) are the velocity coefficients for both air and water phases driven by capillary pressure, combined pressure and gravity, respectively. By solving the cell problems on one of these regularly repeating units, the parameters for Richards' equation can be determined based on the geometry of a single unit, assuming that this is representative of the whole domain *Ω*.

Daly & Roose [7] show that, by assuming that the pressure of the air phase is constant, equation (2.1a) reduces to the saturation form of Richards' equation, assuming $\bar{K}(S)$ and ${\bar{b}}_{g}(S)$ are small enough compared with $\bar{b}(S)$ so that, for constant pressure, equation (2.1b) is approximately satisfied. The relation between equations (1.1) and (2.1a) is discussed further in Daly & Roose [7].

### (c) Model implementation

The parameters for Richards' equation are obtained by solving two sets of problems on the computational mesh created from the segmented geometry of the soil, as described in §2a. In this section, the two problems and how they are implemented are described. The first problem determines the position of the air and water phases within the geometry. The second problem describes the velocities of the air and water so that the parameters of Richards' equation can be calculated by averaging these values. These two sets of problems were solved using COMSOL Multiphysics v. 5.2 (COMSOL AB, Sweden), a commercial finite-element software (table 1).

**Table 1.** Dimensionless parameter values used for the image-based simulations. Where no units are reported, the value is dimensionless. Note that Ca/Pe=1 has been used here to illustrate the model, and that other values of Pe and Ca could be used and would correspond to different length scales. Changing the viscosities of the fluids, i.e. modelling fluids other than air and water, also impacts the values of Pe and Ca.

parameter	value	description
*Ca*/*Pe*	1	ratio of capillary number to Peclet number
*θ*	0°, 20°, 60°	contact angle
*η*^(0)^	2×10^−5^ *Pa* *s*	viscosity of air
*η*^(1)^	1×10^−3^ *Pa* *s*	viscosity of water
*λ*	1×10^−3^	thickness of interface

The first problem solves the leading-order terms for the phase field equations. The fluid–fluid interface location is found by calculating the steady-state solution to

2.6a $$\begin{array}{rl} & \frac{\partial \phi_{0}}{\partial \tau_{-1}}-\frac{\mathrm{Ca}}{\mathrm{Pe}}\nabla \;\nabla_{y}\cdot M_{0}\nabla \;\nabla_{y}\mu_{0}=0,\;y\;y\in B, \end{array}$$

2.6b $$\begin{array}{rl} & \mu_{0}=\frac{f^{\prime }(\phi_{0})}{\lambda }-\lambda \nabla \;\nabla_{y}^{2}\phi_{0},\;y\;y\in B, \end{array}$$

2.6c $$\begin{array}{rl} & \hat{n\;n}\cdot \lambda \nabla \;\nabla_{y}\phi_{0}=-h^{\prime }(\phi_{0}),\;y\;y\in \partial B, \end{array}$$

2.6d $$\begin{array}{rl} \text{and}\; & \hat{n\;n}\cdot M_{0}\nabla \;\nabla_{y}\mu_{0}=0,\;y\;y\in \partial B, \end{array}$$

where an interface of a finite width, λ, is introduced between the two fluids to enable the computation model to solve in finite time. By *τ*_−1_ is denoted the fast time scale, which corresponds to the time taken for the fluid–fluid interface to equilibrate, as opposed to the slow time scale, *τ*_1_, which corresponds to the time required for the saturation to change due to pressure gradients. Effectively, we are making the standard porous media modelling assumption that the fluid inertia is negligible. The Peclet number is

2.7 $$\mathrm{Pe}=\frac{{\tilde{L}}_{x}{\tilde{L}}_{y}\tilde{\zeta }[u\;u]}{\alpha \tilde{\gamma }},$$

where $\tilde{\zeta }$ is the fluid–fluid drag coefficient. Here $M_{0}=\phi_{0}^{2}{\left(1-\phi_{0}\right)}^{2}$, $\hat{n\;n}$ is the unit normal to the soil particle surface, *h*′(*ϕ*_0_) describes the effect of the contact angle, *θ*, between the fluid–fluid interface and solid surfaces, where ′ indicates the functional derivative with respect to *ϕ*, *δ*/*δϕ*. Physically, equation (2.6c) defines the angle between the fluid–fluid interface and the soil particle surfaces; for a small contact angle (approx. 0°) this would correspond to a hydrophilic surface, whereas a large contact angle (greater than 90°) would correspond to a hydrophobic surface. The function $f(\phi_{0})=\phi_{0}^{2}{\left(1-\phi_{0}\right)}^{2}$. The parameter values used for the computational study presented here are summarized in table 1. This set of equations couples together *ϕ*_0_, the leading-order term of the phase field, and *μ*_0_, the leading-order capillary term. To solve this set of equations, it is necessary to define either *ϕ*_0_ or *μ*_0_ in order to determine the corresponding value for the respective variable. Using the model, this can be done in two ways:

— Set the relative water saturation in the geometry and calculate the capillary pressure, *μ*_0_, required to achieve this. The position of the air and water phases at the defined relative water saturation will be dependent on the initial position of the air and water. The contact angle-dependent boundary condition on the soil particles, equation (2.6c), influences the phase field close to the soil particle surface, and this in turn affects the value of the capillary pressure required to hold the water in a particular position.
— Set the capillary pressure and compute the respective phase field. This is similar to the experimental method of applying a pressure and measuring the water content. The advantage of the model presented here is that not only is the water content known, but also the location of the water and air phases within the geometry. This is difficult to observe physically due to the opaque nature of soil.

The values of *μ*_0_ and *ϕ*_0_ vary with respect to time, *τ*_−1_, but it is assumed that the movement of the fluid–fluid interface is the fastest time scale in the model and therefore only the steady state is of interest here.

Note that equations (2.6) are numerically stiff, i.e. there are multiple different time scales over which the variables are changing [26], due to the presence of *M*_0_. At steady state, *μ*_0_ is constant and independent of *M*_0_. As only the steady-state solution is of interest here, neglecting *M*_0_ does not affect the final solution, only how it is computed. Therefore, *M*_0_ is neglected in order to increase efficiency and the following equations are solved:

2.8a $$\begin{array}{rlr} & \frac{\partial \phi_{0}}{\partial \tau_{-1}}-\frac{\mathrm{Ca}}{\mathrm{Pe}}\nabla \;\nabla_{y}^{2}\mu_{0}=0, & \;y\;y\in B, \end{array}$$

2.8b $$\begin{array}{rlr} & \mu_{0}=\lambda^{-1}f^{\prime }(\phi_{0})-\lambda \nabla \;\nabla_{y}^{2}\phi_{0}, & \;y\;y\in B, \end{array}$$

2.8c $$\begin{array}{rlr} & \hat{n\;n}\cdot \lambda \nabla \;\nabla_{y}\phi_{0}=-h^{\prime }(\phi_{0}), & \;y\;y\in \partial B, \end{array}$$

2.8d $$\begin{array}{rlr} \text{and}\; & \hat{n\;n}\cdot \nabla \;\nabla_{y}\mu_{0}=0, & \;y\;y\in \partial B. \end{array}$$

If *μ*_0_ satisfies equations (2.8), then it will also satisfy the original equations (2.6). The new form used here has the advantage of being less stiff and more computationally efficient.

The equations presented here are reformulated from Daly & Roose [7] in order to improve the numerical stability, reduce stiffness and make the finite-element model more efficient. These minor modifications correspond to an altered initial free energy used in Daly & Roose [7]. There is still ambiguity in the theoretical physics community about the precise functional form of the free energy, and any sensible formulation could be used as an input for the Cahn–Hilliard equation for the two-fluid expression. Although in using the Cahn–Hilliard equations it is stated that the value of *ϕ*=0 for fluid 0 and *ϕ*=1 for fluid 1, and that the values in between correspond to the interface of finite thickness between the two fluids, it is possible for *ϕ* to have values of 1±λ by the nature of the equations. The altered equations presented in the following paragraphs, which overcome this issue, could be obtained using the procedure presented by Daly & Roose [7] or used as inputs to the procedure, and would result in the same formulation of Richards' equation. Firstly, additional terms were added to the boundary condition expression involving the contact angle, *h*′(*ϕ*_0_), presented in Daly & Roose [7]. This is necessary to prevent large values of *ϕ*_0_ occurring at the edges of the geometry and to improve the stability of the fluid flow model,

2.9 $$\begin{array}{rl} h^{\prime }(\phi_{0}) & \;=\underset{\mathrm{original}\;[7]}{\underbrace{\sqrt{2}\cos(\theta )\phi_{0}(1-\phi_{0})}}\\ & \;\;+\underset{\mathrm{additional}\;\mathrm{terms}}{\underbrace{0.5\left(1+\tanh\left(-\frac{\phi_{0}}{\lambda }\right)\right)\phi_{0}^{2}\cos(\theta )-0.5\left(1+\tanh\left(-\frac{1-\phi_{0}}{\lambda }\right)\right){\left(1-\phi_{0}\right)}^{2}\cos(\theta )}}. \end{array}$$

**Figure 3.**
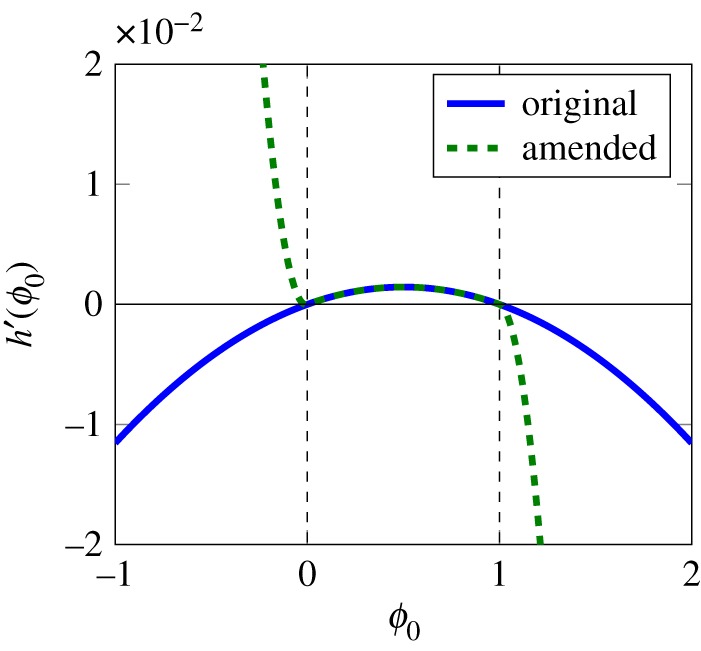
The original contact angle condition from Daly & Roose [7] compared with the amended condition, equation (2.9). The dashed green lines indicate the interval of interest where *ϕ*∈[0,1]. It can be seen that within this region the two conditions are the same. (Online version in colour.)

This does not change the contact angle in the region of interest, i.e. *ϕ*∈[0,1], as shown in figure 3.

Secondly, the numerical model formulation of the free energy previously used allows *ϕ*_0_ to become slightly negative, which does not have a physical interpretation. To prevent negative viscosity values, the viscosity variation between the two fluids was implemented using

2.10 $$\eta_{0}=\frac{\eta^{\left(0\right)}}{\eta^{\left(1\right)}}+\frac{\eta^{\left(1\right)}-\eta^{\left(0\right)}}{\eta^{\left(1\right)}}\min\;(1,\max(\phi_{0},0)).$$

The solution to the phase field equations (2.8) was used as an input to the cell problems presented below. The cell problems are derived as part of the homogenization procedure, which is described by Daly & Roose [7]. The equations calculate the first non-zero velocity term due to external perturbations, such as capillary pressure gradients and other body forces, the first non-zero pressure term and the first-order correction to the capillary pressure and phase field. It should be noted that the correction to the capillary term scales with λ and hence becomes 0 for an infinitely thin interface, and that it is not necessary to formally calculate the correction to the phase field.

To improve the numerical stability of the cell problems, the equations presented in Daly & Roose [7] were reformulated to define ${\bar{\omega }}_{k}^{\mu }=\omega_{k}^{\mu }+\phi_{0}\chi_{k}^{\mu }$ and ${\bar{\omega }}_{k}^{p}=\omega_{p}^{\mu }+\phi_{0}\chi_{p}^{\mu }$, where $\omega_{k}^{\mu }$ and $\chi_{k}^{\mu }$ are local variations in the combined pressure and capillary pressure, respectively, caused by the macroscale capillary pressure. We see that $\omega_{k}^{p}$ and $\chi_{k}^{p}$ are local variations in the combined pressure and capillary pressure, respectively, caused by the macroscale combined pressure. Also, the equations have been rescaled so that there is only one dimensionless coefficient. Thus, in order to calculate $\kappa \;\kappa_{k}^{\mu }$ and *κ*^*μ*^_*p*_, the cell problems were implemented as

2.11a $$\begin{array}{rlr} & \kappa \;\kappa_{k}^{\mu }\cdot \nabla \;\nabla_{y}\phi_{0}-\frac{\mathrm{Ca}}{\mathrm{Pe}}[\nabla \;\nabla_{y}\cdot M_{0}\nabla \;\nabla_{y}\chi_{k}^{\mu }+\nabla \;\nabla_{y}\cdot M_{0}{\hat{e\;e}}_{k}]=0, & \;y\;y\in B, \end{array}$$

2.11b $$\begin{array}{rlr} & \nabla \;\nabla_{y}\cdot \sigma_{k}^{\mu }-\nabla \;\nabla_{y}{\bar{\omega }}_{k}^{\mu }-\chi_{k}^{\mu }\nabla \;\nabla_{y}\phi_{0}=\phi_{0}{\hat{e\;e}}_{k}, & \;y\;y\in B, \end{array}$$

2.11c $$\begin{array}{rlr} & \nabla \;\nabla_{y}\cdot \kappa \;\kappa_{k}^{\mu }=0, & \;y\;y\in B, \end{array}$$

2.11d $$\begin{array}{rlr} & \kappa \;\kappa_{k}^{\mu }=0,\; & y\;y\in \partial B, \end{array}$$

2.11e $$\begin{array}{rlr} \text{and}\; & \hat{n\;n}\cdot M_{0}\nabla \;\nabla_{y}\chi_{k}^{\mu }+\hat{n\;n}\cdot M_{0}{\hat{e\;e}}_{k}=0, & \;y\;y\in \partial B, \end{array}$$

where $\sigma_{k}^{\mu }=\nabla \;\nabla_{y}\kappa \;\kappa_{k}^{\mu }+{\left(\nabla \;\nabla_{y}\kappa \;\kappa_{k}^{\mu }\right)}^{T}$ is the local variation in the stress tensor driven by the capillary pressure, and

2.12a $$\begin{array}{rlr} & \kappa \;\kappa_{k}^{p}\cdot \nabla \;\nabla_{y}\phi_{0}-\frac{\mathrm{Ca}}{\mathrm{Pe}}\nabla \;\nabla_{y}\cdot M_{0}\nabla \;\nabla_{y}\chi_{k}^{p}=0, & \;y\;y\in B, \end{array}$$

2.12b $$\begin{array}{rlr} & \nabla \;\nabla_{y}\cdot \sigma_{k}^{p}-\nabla \;\nabla_{y}{\bar{\omega }}_{k}^{p}-\chi_{k}^{p}\nabla \;\nabla_{y}\phi_{0}={\hat{e\;e}}_{k}, & \;y\;y\in B, \end{array}$$

2.12c $$\begin{array}{rlr} & \nabla \;\nabla_{y}\cdot \kappa \;\kappa_{k}^{p}=0, & \;y\;y\in B, \end{array}$$

2.12d $$\begin{array}{rlr} & \kappa \;\kappa_{k}^{p}=0, & \;y\;y\in \partial B, \end{array}$$

2.12e $$\begin{array}{rlr} \text{and}\; & \hat{n\;n}\cdot M_{0}\nabla \;\nabla_{y}\chi_{k}^{p}=0, & \;y\;y\in \partial B, \end{array}$$

where $\sigma_{k}^{p}=\nabla \;\nabla_{y}\kappa \;\kappa_{k}^{p}+{\left(\nabla \;\nabla_{y}\kappa \;\kappa_{k}^{p}\right)}^{T}$ is the local variation in the stress tensor driven by the combined pressure. For a small enough interface width $\kappa \;\kappa^{g}=\kappa \;\kappa_{3}^{\mu }$ [7].

A requirement of the above model is that the porous domain is made up of regularly repeating units, i.e. the structure is periodic. This is not the case for real soil samples. The periodicity is introduced by reflecting the cubic geometry in three sides, one in each of the *x*, *y* and *z* directions. This is done mathematically by introducing symmetric boundary conditions on the outer boundaries of the fluid domain. This results in the cubic geometry being effectively eight times larger. The boundary conditions are summarized in tables 2 and 3.

**Table 2.** Symmetric boundary conditions for the phase field, equations (2.8). *ϕ*_0_ is the leading order of the phase field and *μ*_0_ is the leading order of the capillary pressure.

*x*=0, 0.5	*y*=0, 0.5	*z*=0, 0.5
∂_*x*_*ϕ*_0_=0	∂_*y*_*ϕ*_0_=0	∂_*z*_*ϕ*_0_=0
∂_*x*_*μ*_0_=0	∂_*y*_*μ*_0_=0	∂_*z*_*μ*_0_=0

**Table 3.** Symmetric boundary conditions for cell order problems, equations (2.11), (2.12) where *j*=*μ*,*p*. *k*=1,2,3 corresponds to the direction in which the body force is being applied, $u_{k}^{j}$, $v_{k}^{j}$ and $w_{k}^{j}$ are the components of the velocity vector $\kappa_{k}^{j}$, $\omega_{k}^{j}$ is the first non-zero term of the combined pressure and $\chi_{k}^{j}$ is the first-order correction to the capillary pressure.

	*x*=0,0.5	*y*=0,0.5	*z*=0,0.5
*k*=1	$\partial_{x}u_{1}^{\;j}=0$	$\partial_{y}u_{1}^{\;j}=0$	$\partial_{z}u_{1}^{\;j}=0$
	$v_{1}^{\;j}=0$	$v_{1}^{\;j}=0$	$\partial_{z}v_{1}^{\;j}=0$
	$w_{1}^{\;j}=0$	$\partial_{y}w_{1}^{\;j}=0$	$w_{1}^{\;j}=0$
	$\omega_{1}^{\;j}=0$	$\partial_{y}\omega_{1}^{\;j}=0$	$\partial_{z}\omega_{1}^{\;j}=0$
	$\chi_{1}^{\;j}=0$	$\partial_{y}\chi_{1}^{\;j}=0$	$\partial_{z}\chi_{1}^{\;j}=0$
*k*=2	$u_{2}^{\;j}=0$	$u_{2}^{\;j}=0$	$\partial_{z}u_{2}^{\;j}=0$
	$\partial_{x}v_{2}^{\;j}=0$	$\partial_{y}v_{2}^{\;j}=0$	$\partial_{z}v_{2}^{\;j}=0$
	$\partial_{x}w_{2}^{\;j}=0$	$w_{2}^{\;j}=0$	$w_{2}^{\;j}=0$
	$\partial_{x}\omega_{2}^{\;j}=0$	$\omega_{2}^{\;j}=0$	$\partial_{z}\omega_{2}^{\;j}=0$
	$\partial_{x}\chi_{2}^{\;j}=0$	$\chi_{2}^{\;j}=0$	$\partial_{z}\chi_{2}^{\;j}=0$
*k*=3	$u_{3}^{\;j}=0$	$\partial_{y}u_{3}^{\;j}=0$	$u_{3}^{\;j}=0$
	$\partial_{x}v_{3}^{\;j}=0$	$v_{3}^{\;j}=0$	$v_{3}^{\;j}=0$
	$\partial_{x}w_{3}^{\;j}=0$	$\partial_{y}w_{3}^{\;j}=0$	$\partial_{z}w_{3}^{\;j}=0$
	$\partial_{x}\omega_{3}^{\;j}=0$	$\partial_{y}\omega_{3}^{\;j}=0$	$\omega_{3}^{\;j}=0$
	$\partial_{x}\chi_{3}^{\;j}=0$	$\partial_{y}\chi_{3}^{\;j}=0$	$\chi_{3}^{\;j}=0$

Enforcing periodicity on the soil sample means that the geometry for the numerical model no longer represents the imaged soil. However, assuming the soil is isotropic, the errors induced by this assumption exist only on the boundaries of the domain. Hence, as the size of the domain increases, the relative contribution of this error will decrease and the volume becomes representative [13]. The method for ensuring that the representative elementary volume (REV) is large enough is described in §2d. This is an approach that has been used in previous studies [27,21].

### (d) Representative elementary volume

The microscale unit has to be representative of the macroscale geometry. To determine the size of the REV, increasing sizes of the microscale geometry were modelled. To find a suitable unit size (${\tilde{L}}_{y}$), five cubes were used with increasing side lengths: 0.114 mm, 0.234 mm, 0.354 mm, 0.474 mm and 0.594 mm. These sizes were selected as they create cubes with an integer number of voxels. The meshes were all created from the centre of the image stack where the image quality is best. These meshes were used to calculate the wetting and drying curves by solving equations (2.8). The initial condition for each saturation was taken from the previous saturation, except for the first model run where the initial condition was set manually. The saturation was increased or decreased in steps of 1%. The wetting and drying curves were calculated by fixing the saturation value and running the model to steady state to evaluate the corresponding capillary pressure. If the wetting and drying curves did not form a closed loop, i.e. the wetting and drying behaved differently at high or low water potentials, it was assumed that the manually set initial conditions were inaccurate and either the wetting or drying curve was recalculated. The percentage difference of the wetting curves and drying curves between each of the five cubes and the cube with length size 0.594 mm was calculated using

2.13 $$E={\left(\sum_{i=n}^{N}{\left(\frac{2(v_{i}-u_{i})}{\left(v_{i}+u_{i}\right)}\right)}^{2}\right)}^{1/2}\times \frac{100}{N-n}.$$

Here, *n* is the minimum saturation, in this case 20%, *N* is the maximum saturation, in this case 80%, *v*_*i*_ is the capillary pressure for saturation *i* of each different cube size and *u*_*i*_ is the capillary pressure for saturation *i* for the cube with length size 0.594 mm. A fully wetted condition, i.e. *θ*=0, was used for the boundary condition.

After selecting the appropriate geometry size, a mesh refinement study was carried out using meshes created in ScanIP. Initially, a mesh is generated based on the resolution of the images, with maximum edge length 6 μm and minimum edge length 3 μm (559 169 elements); then this mesh is coarsened by increasing the element edge lengths. The coarseness was initially decreased in steps of −5 until the coarseness setting of −10 and then in steps of −10 until the coarseness setting of −50 (edge lengths: max. 60 μm, min. 24 μm, 66 672 elements) was reached. The mesh coarseness for all subsequent models was chosen when the percentage difference between the resulting values for $\kappa \;\kappa_{k}^{p}$ was less than 5%, as with the REV study.

### (e) Soil–water characteristic curve

After selecting the appropriate REV and mesh size, the capillary pressures were calculated for every 1% saturation between 2% and 95%. The mesh was used to calculate the soil–water retention curve for the imaged soil sample for fully wetted soil particle surfaces, contact angle 0°, and also contact angles of 20° and 60°. The full set of equations, (2.8), was used for the representative microscale unit size, mesh refinement study and to calculate the 20° and 60° contact angle wetting and drying curves. For the fully wetted surface model the set of equations was reduced to

2.14a $$\begin{array}{rl} & \frac{\partial \phi_{0}}{\partial \tau }=f^{\prime }(\phi_{0})-\lambda^{2}\nabla_{y}^{2}\phi_{0}-\lambda \mu_{0},\;y\;y\in B, \end{array}$$

2.14b $$\begin{array}{rl} & \phi_{0}=1,\;y\;y\in \partial B, \end{array}$$

2.14c $$\begin{array}{rl} \text{and}\; & \;\int_{B}\phi_{0}\;dy\;y=S\int_{B}1\;dy\;y. \end{array}$$

The value of *S* is specified. These equations are sufficient for calculating *ϕ*_0_ and *μ*_0_ with the advantage that they are faster and require less memory to compute. The addition of the ∂*ϕ*_0_/∂*t* term to equation (2.14a) forces a unique *μ*_0_ for a given initial condition. The model is run from the results of the previous saturation to steady state. We note that to calculate the dimensional capillary pressure, $\tilde{\mu }$, it is necessary to use the scaling

2.15 $$\tilde{\mu }=\frac{\alpha \tilde{\gamma }}{{\tilde{L}}_{y}(N-1)}\mu_{0},$$

where *N* is the number of dimensions.

The parameters for Richards' equation were calculated for each saturation. To do this, firstly, the phase field equations (2.8) were computed on the Iridis 4 Supercomputing cluster at the University of Southampton, UK, using the batch nodes (16 processors, up to 64 GB RAM) which used 15 min to 5 h of run time and 7 GB of RAM. The phase field calculations had to be evaluated in series as each saturation used the solution for the previous saturation as the initial condition. Secondly, three further calculations for the three-directional components of each of the two cell problems, equations (2.11) and (2.12), were calculated on the Iridis 4 Supercomputing cluster on the high-memory nodes (32 processors, up to 256 GB RAM) and on two bespoke high-memory desktops (24 processors, up to 512 GB RAM, 16 processors, up to 768 GB RAM) using 45 min to 3 h run time and 160 GB of RAM.

## 3. Results

### (a) Representative microscale unit size

**Figure 4.**
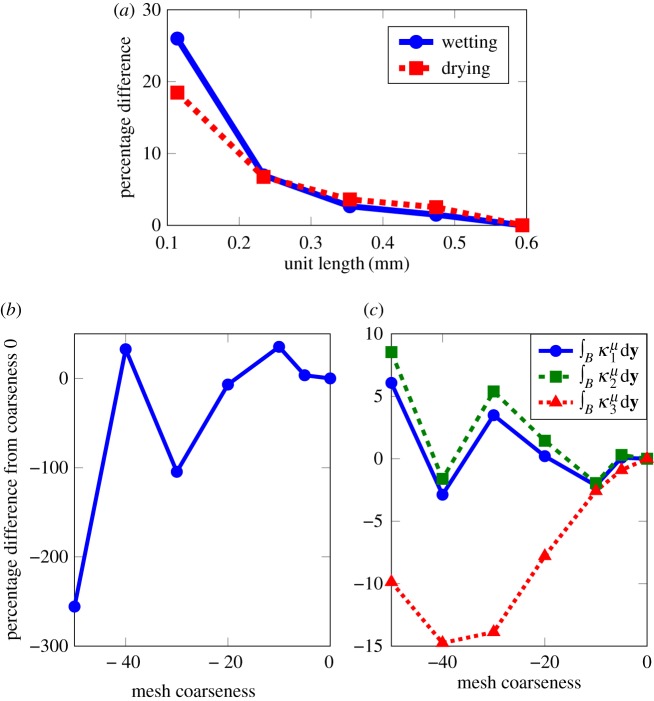
The percentage differences were used to select appropriate REV and mesh requirements. Mesh coarseness relates to the maximum and minimum element edge lengths of the mesh, e.g. mesh coarseness 0: max. 6 μm, min. 3 μm (559 169 elements) and mesh coarseness −50: max. 60 μm, min. 24 μm (66 672 elements). (*a*) The percentage difference, as given by equation (2.13), for the wetting curves and drying curves between 20% and 80% saturation from unit length 0.594 mm. (*b*) Mesh refinement results for capillary pressure and (*c*) mesh refinement study results for $\bar{K}(S)$. (Online version in colour.)

 Figure 4*a* illustrates the model output of the percentage difference of wetting and drying curves between the different cube sizes and the largest cube size. It was decided that the unit with side length 0.474 mm would be used as the results were less than 5% different from those of the larger unit with length size 0.594 mm, but took between 30 min and 3 h per saturation value compared with the larger unit size, which took between 5 h and 25 h per saturation value.

The results of the mesh refinement study are shown in figure 4*b*,*c*. A mesh coarseness of −5 is considered acceptable for the modelling purposes presented here, because the percentage difference between two models was less than 5%. The final mesh had a boundary layer of 0.002 mm, 75 0955 elements, with maximum edge size 0.0359 mm and target minimum edge size 0.0051 mm.

### (b) Soil–water characteristic curve

**Figure 5.**
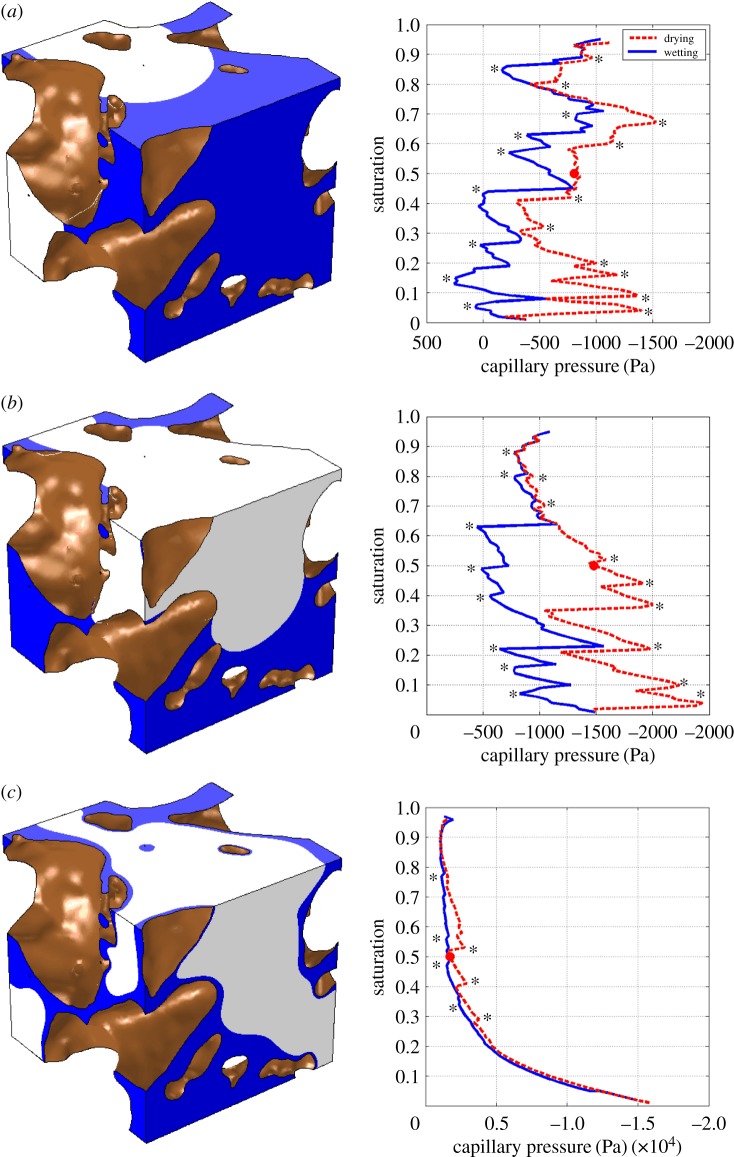
Wetting and drying curves produced from the phase field equations (2.8) and (2.14). A representative image from the simulation at 50% saturation, corresponding to the circle on the graph, is also shown to highlight the difference that the contact angle makes to the interface position. The water is shown in blue and the air in white, and browndenotes the surface of the soil particles. The SWCC exhibits Haines' jump effects, some of which are highlighted by asterisks. Videos of the soil units wetting and drying can be found in the electronic supplementary material online. (*a*) 60° contact angle, (*b*) 20° contact angle and (*c*) fully wetted, 0° contact angle. (Online version in colour.)

The SWCCs for the three contact angle cases can be seen in figure 5. The curves all show hysteresis effects with different capillary pressures required to wet or dry the soil. Haines' jumps occur in all three contact angle cases. Higher contact angles cause greater hysteresis effects and more Haines' jumps than the fully wetted condition (figure 5). The contact angles of 60° and 20° produce SWCCs that drain at much smaller pressures than the 0° case. There are also more Haines' jumps and larger hysteresis loops than the fully wetted surface condition. The fully wetted condition, 0° contact angle, shows the negative gradient and plateau of the intrinsic shape of the SWCC, but not the plateau at high saturations.

**Figure 6.**
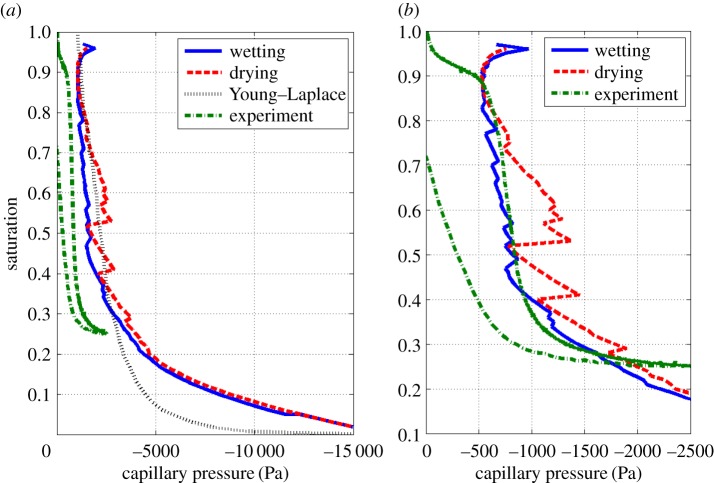
Comparison of experimentally measured and computed SWCCs. (*a*) Comparison of model results with the Young–Laplace approximation and experimental data. (*b*) Experimental data compared with model capillary pressures scaled by a factor of 2. (Online version in colour.)

In figure 6*a*, the SWCC for the 0° case is compared with the Young–Laplace model calculated from the image stack used for the modelling and the results of the experimental study (see the electronic supplementary material for experimental methods). The experiment was carried out *a priori* on a larger soil sample than was imaged. The imaged soil sample and experimental soil sample had a porosity of 0.6 and 0.62, respectively. The Haines' jumps present in the model results are highly geometry dependent and, as it was not possible to use exactly the same geometries for testing, it is not unexpected that Haines' jumps are invisible in the experimental data.

The model shows good agreement with the Young–Laplace equation, particularly at high water potentials. The capillary pressure calculated by the model is different by approximately a factor of 2 compared with the experimental data. This would mean that, by halving the surface tension used to dimensionalize the capillary pressure, the model and experimental results could be brought closer together, as shown in figure 6*b*. This factor of 2 difference between the experiment and model results could also be related to the assumption in the model that there is only pure water within the soil. However, in the experiment there would be a soil solution, rather than a pure water phase. This means that the contact angle and surface tension values are most probably affected. Further, the contact angle of the air–soil solution interface with the soil particles is unknown and, as can be seen by comparing the 0° and 20° contact angle results, higher contact angles decrease the capillary pressure required to drain the soil. Full validation of the model would require high-precision measurements of all these properties, which would be novel in themselves; this is outside the scope of the current paper.

### (c) Parameters for Richards' equation

**Figure 7.**
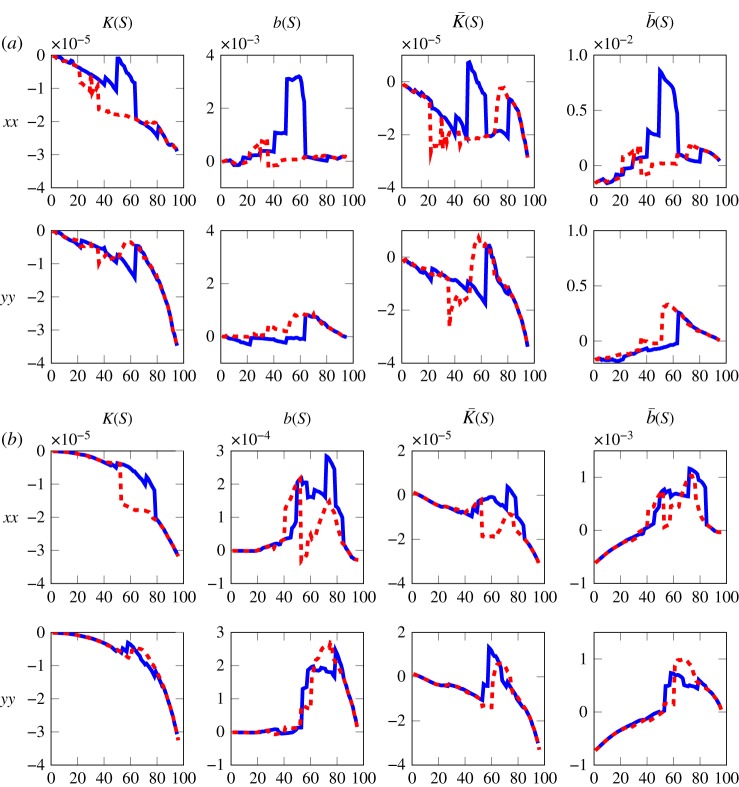
Components of *K*(*S*), *b*(*S*), $\bar{K}(S)$ and $\bar{b}(S)$ defined in equations (2.4). *S* is the relative water saturation of the pore space, and *K*(*S*) and *b*(*S*) are the average velocities of the water for a unit drop in capillary and combined pressure, respectively. $\bar{K}(S)$ and $\bar{b}(S)$ are the average velocities of both the water and the air for a unit drop in capillary and combined pressure, respectively. Full results can be found in the electronic supplementary material. (*a*) Contact angle 20° and (*b*) contact angle 0°. (Online version in colour.)

The parameters for Richards' equation are calculated using equations (2.4). To assist with the analysis of the results, a summary of the parameter descriptions is presented in table 4. Figure 7 shows the diagonal elements of the tensor parameters of Richards' equation calculated using the model with 20° and 0° contact angles. Note that, by symmetry, the off-diagonal elements of *K*(*S*), *b*(*S*), $\bar{K}(S)$ and $\bar{b}(S)$ are 0. The graphs reflect the behaviour of the SWCC results, with the results for 20° being less monotonic than the results for 0°, and the parameters show similar trends to the results of an idealized geometry in Daly & Roose [7]. We see that *a*(*S*)*K*(*S*), in equation (2.1a), takes the place of the soil moisture diffusivity in equation (1.1). From the results presented here, neglecting Haines' jumps, *a*(*S*) and *K*(*S*) are both negative, therefore *a*(*S*)*K*(*S*) is positive. This agrees with the expected value for the soil moisture diffusivity, which would be positive.

**Table 4.** Description of Richards' equation parameters in the form derived by Daly & Roose [7].

parameter	description
*S*	relative water saturation of the pore space
*a*(*S*)	gradient of capillary pressure with respect to saturation
*K*(*S*)	average of water velocity driven by capillary pressure
*b*(*S*)	average of water velocity driven by combined pressure
$\bar{K}(S)$	average of both water and air velocities driven by capillary pressure
$\bar{b}(S)$	average of both water and air velocities driven by combined pressure

**Figure 8.**
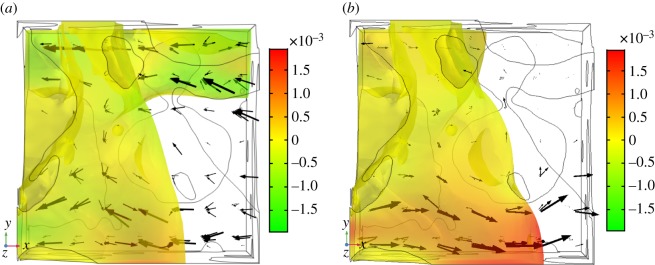
Air phase velocity (m s^−1^) either side of Haines' jumps that cause $\bar{K}(S)$ to be positive. Note that the water phase and soil particles are not coloured so that the topology of the airphase can be displayed clearly. The grey lines show the outline of the soil geometry, the black arrows show the direction of flow in both the water and air phases and are scaled with the velocity magnitude. The axes indicate the positive direction. These figures are from the 20° contact angle case. (*a*) Before wetting: saturation 49% and (*b*) after wetting: saturation 50%. (Online version in colour.)

There are some values of $\bar{K}(S)$ which are positive. The reason for this is demonstrated in figure 8. Figure 8*a* shows the air phase, coloured by the velocity value, at 49% saturation during the wetting process. Note that the water phase is not plotted so that the topological differences between the figures can be clearly demonstrated. To calculate $\bar{K}(S)$, the water phase is being driven by the capillary pressure in the negative *x*-direction; see the right-hand side of equation (2.11b). This produces a velocity in the water, which in turn produces a velocity in the air phase. The arrows in figure 8*a* show the direction of flow in both the water and air phases, the size being scaled by the velocity magnitude; the flow in both the water and air phases is predominately in the same direction as the applied force, i.e. the negative *x*-direction. Therefore, the value of $\bar{K}(S)$, calculated by integrating the velocities of both the air and water phases over the microscale domain, is negative. In figure 8*a*, the air phase is connected in the *x*-plane, i.e. the air phase crosses the whole unit volume. However, in figure 8*b*, which shows the air phase after the saturation has been increased by 1%, it can be seen that a Haines' jump has occurred, i.e. the topological configurations between figure 8*a*,*b* are different. The air phase is no longer connected in the *x*-plane and the dominant direction of flow in the air has changed direction to the positive *x*-direction. The air velocity is often higher than the water velocity as it is less viscous, and hence when integrating the velocities of both the air and water phases the air velocity dominates, resulting in a positive value for $\bar{K}(S)$.

The positive values occur at Haines' jumps, where the air phase is not connected in the plane parallel to the direction of the driven fluid. This is demonstrated in figure 8, where the velocity of the air has been plotted with the unit cube. For the 20° contact angle case the value of $\bar{b}(S)$ is one to two orders of magnitude larger than $\bar{K}(S)$ apart from saturations between 37–41% and 95%. At these saturations, $\bar{b}(S)$ would dominate the flow behaviour and therefore a constant fluid pressure could be assumed. This assumption reduces equation (2.1a) to the saturation form of Richards' equation, as discussed in Daly & Roose [7]. This is also true for the 0° case, for saturations in the range 55–85%.

The level of anisotropy between the *xx*, *yy* and *zz* directions for *K*(*S*) and $\bar{K}(S)$ (see the electronic supplementary material for full results) does not appear to be very great. This implies that it may be possible to estimate the anisotropic tensors with isotropic ones. However, the soil image to create the geometry for the model had been prepared with the aim of making the sample as homogeneous as possible. Whether or not one can approximate the anisotropic tensors with isotropic ones will depend on the isotropy of the underlying geometry and the angle of the samples with respect to the coordinate axes.

## 4. Discussion

We have used the model of Daly & Roose [7] to investigate the influence of contact angle on pore-scale water retention and flow in real soil structures obtained using micrometre-scale non-invasive 3D imaging. The effect on the SWCC of using three different contact angles has been investigated. It is possible to model experimentally observed physical behaviour such as Haines' jumps. The jumps occur when a pore rapidly drains or fills. This can be visualized in the model results and gives direct insight into how the water moves in a specific soil sample. The calculated SWCC was then used to calculate parameters for Richards' equation, which were also found to exhibit hysteresis and ‘jumping’ behaviour.

It has been shown with this model and other computational models of multi-phase flow in soil [28,29] that, although various different contact angles have been measured experimentally, a 0° contact angle gives the closest simulation to the intrinsic shape of the SWCC. It is particularly unlikely that the SWCC resulting from a contact angle of 60° is realistic as it requires higher capillary pressures to drain very high saturations compared with low saturations.

Image-based modelling can only be as accurate as the images that are used to create the geometry. In this case, the resolution of the images means that pores less than approximately 6 μm are not resolved and hence are not captured by the model. The pressure difference, Δ*p*, required to drain a pore of radius *a* is described by the Young–Laplace equation

4.1 $$\Delta p=\frac{2\gamma \cos\theta }{a}.$$

This gives pressure differences of 48, 45.1 and 24 kPa for 0°, 20° and 60° contact angles, respectively, which are required to drain pores with a diameter of 6 μm. These pressure differences are similar to conditions in a freely drained field soil. As the capillary pressures calculated for the 60° and 20° contact angles are below their respective values, the results presented in figure 5*a*,*b* would be affected by smaller pores. Including the small pores would increase the value of the saturation for the range of capillary pressure presented in figure 5. A standard problem, when assuming that the position of the interface is mainly determined by capillary forces, is that an infinite pressure would be required to completely wet or dry the soil. Therefore, the accuracy of the model at high and low water potentials requires further investigation. A further limitation is that the soil particles are assumed solid (akin to individual sand grains). The inter-aggregate pores would contribute to the SWCC and the pore structure between aggregates will change due to cycles of wetting and drying or overburden stresses [30]. Our modelling approach could be applied to more realistic pore structures, but intact specimens were not used for this study to allow for repeatable high-resolution scanning and subsequent model development. The main bottleneck of the current method is the time required to complete the computations for each saturation. The total computational time in this first-of-a-kind study was four weeks using a supercomputer and high-memory bespoke desktops.

Full validation of this model with experiments is required to determine the accuracy of the method. Validation would require the SWCC and hydraulic conductivity tensor to be measured at multiple saturations and for the hydraulic conductivity to be measured in all three directions for the exact same soil sample that was imaged. This would mean conducting the measurements on a soil sample with the 6 mm diameter syringe barrel used for imaging. This high-precision experimental set-up would in itself be highly novel and is outside the scope of the current paper.

The results are very sensitive to the contact angle. The 60° contact angle gave results that are unlikely to be realistic despite being within the range measured experimentally [14]. At the microscale conditions of the model, however, contact angles can be less than half the values measured with traditional approaches due to surface roughness impacts. The results for the 20° case show that despite being well below the limit defined as hydrophobic, i.e. less than 90°, the model is exhibiting hydrophobicity, in the context of water flow and retention in soil. The contact angle can be altered due to the presence of plant and microbial exudates in the soil. Carminati [31] showed that the soil around the roots wetted slower around older root segments than newer segments. This could be due to a change in pore size distribution or higher quantities of root exudates around older roots that has increased the contact angle, causing it to rewet more slowly. The 0° contact angle gives the SWCC with the most conventional shape. The 0° contact angle causes the presence of a film of water on the surface of the soil particles at all times. This could be capturing real soil behaviour that is not accounted for in the physics of the Cahn–Hilliard–Stokes model derived in Daly & Roose [7], such as adhesive forces being involved in the initial wetting process [1] and unresolved pore space less than 6 μm that will also influence the SWCC.

## 5. Conclusion

Using the model of Richards' equation from Daly & Roose [7] with image-based geometry, it has been shown that the hydraulic properties of a soil are strongly related to the geometry and contact angle of the fluid–fluid interface and the soil particle surfaces. Larger contact angles lead to more hysteresis of pore water retention between wetting and drying and increased numbers of Haines' jumps. The application of a 0° contact angle still resulted in Haines' jumps and hysteresis that are related to the underlying geometry. It has been shown that it is possible to parameterize Richards' equation for a specific soil using the model and it provides more detailed information in comparison with what is realistically achievable experimentally. In the future, this model could be applied to investigate how plants affect the fluid flow in soil by investigating the effects of exudates on the properties of water. This would improve our fundamental understanding of water movement in the soil and its uptake by plants, leading to improvements in agricultural practices.

## Supplementary Material

Supplementary Text

## Supplementary Material

Supplementary Figure 1

## Data accessibility

Data supporting this study are available on request from the University of Southampton repository at http://dx.doi.org/10.5258/SOTON/405744 [32].

## References

[1] Richards LA 1931 Capillary conduction of liquids through porous mediums. *Physics* 1, 318–333. (doi:10.1063/1.1745010)

[2] Roose T, Fowler A 2004 A model for water uptake by plant roots. *J. Theor. Biol.* 228, 155–171. (doi:10.1016/j.jtbi.2003.12.012) 15094012 10.1016/j.jtbi.2003.12.012

[3] Van Genuchten MT 1980 A closed-form equation for predicting the hydraulic conductivity of unsaturated soils. *Soil Sci. Soc. Am. J.* 44, 892–898. (doi:10.2136/sssaj1980.03615995004400050002x)

[4] Brooks R, Corey A 1964 *Hydraulic properties of porous media*, vol. 3 Fort Collins, CO: Colorado State University.

[5] Nuth M, Laloui L 2008 Advances in modelling hysteretic water retention curve in deformable soils. *Comput. Geotech.* 35, 835–844. (doi:10.1016/j.compgeo.2008.08.001)

[6] Haines WB 1930 Studies in the physical properties of soils. v. The hysteresis effect in capillary properties, and the modes of water distribution associated therewith. *J. Agric. Sci.* 20, 97–116. (doi:10.1017/S002185960008864X)

[7] Daly KR, Roose T 2015 Homogenization of two fluid flow in porous media. *Proc. R. Soc. A* 471, 20140564 (doi:10.1098/rspa.2014.0564) 27547073 10.1098/rspa.2014.0564 PMC4991259

[8] Cahn JW, Hilliard JE 1958 Free energy of a nonuniform system. I. Interfacial free energy. *J. Chem. Phys.* 28, 258–267. (doi:10.1063/1.1744102)

[9] Cahn JW 1959 Free energy of a nonuniform system. II. Thermodynamic basis. *J. Chem. Phys.* 30, 1121–1124. (doi:10.1063/1.1730145)

[10] Cahn JW, Hilliard JE 1959 Free energy of a nonuniform system. III. Nucleation in a two-component incompressible fluid. *J. Chem. Phys.* 31, 688–699. (doi:10.1063/1.1730447)

[11] Pavliotis GA, Stuart AM 2000 *Multiscale methods averaging and homogenization*. New York, NY: Springer.

[12] Arbogast T, Lehr H 2006 Homogenization of a Darcy–Stokes system modeling vuggy porous media. *Comput. Geosci.* 10, 291–302. (doi:10.1007/s10596-006-9024-8)

[13] Daly K, Mooney S, Bennett M, Crout N, Roose T, Tracy S 2015 Assessing the influence of the rhizosphere on soil hydraulic properties using X-ray computed tomography and numerical modelling. *J. Exp. Bot.* 66, 2305–2314. (doi:10.1093/jxb/eru509) 25740922 10.1093/jxb/eru509 PMC4407651

[14] Czachor H, Hallett P, Lichner L, Jozefaciuk G 2013 Pore shape and organic compounds drive major changes in the hydrological characterisitcs of agricultural soils. *Eur. J. Soil Sci.* 64, 334–344. (doi:10.1111/ejss.12052)

[15] McHale G, Newton MI, Shirtcliffe NJ 2005 Water-repellent soil and its relationship to granularity, surface roughness and hydrophobicity: a materials science view. *Eur. J. Soil Sci.* 56, 445–452. (doi:10.1111/j.1365-2389.2004.00683.x)

[16] Bachmann J, McHale G 2009 Superhydrophobic surfaces: a model approach to predict contact angle and surface energy of soil particles. *Eur. J. Soil Sci.* 60, 420–430. (doi:10.1111/j.1365-2389.2008.01118.x)

[17] Philip JR 1971 Limitations on scaling by contact angle. *Soil Sci. Soc. Am. J.* 35, 507–509. (doi:10.2136/sssaj1971.03615995003500030048x)

[18] Lourenço SDN, Woche SK, Bachmann J, Saulick Y 2015 Wettability of crushed air-dried minerals. *Geotech. Lett. 2015* 5, 173–177. (doi:10.1680/jgele.15.00075)

[19] Tahmasebi P, Javadpour F, Sahimi M 2015 Multiscale and multiresolution modeling of shales and their flow and morphological properties. *Sci. Rep.* 5, 16373 (doi:10.1038/srep16373) 26560178 10.1038/srep16373 PMC4642334

[20] Tahmasebi P, Javadpour F, Sahimi M, Piri M 2016 Multiscale study for stochastic characterization of shale samples. *Adv. Water Resour.* 89, 91–103. (doi:10.1016/j.advwatres.2016.01.008)

[21] Daly KR, Keyes SD, Masum S, Roose T 2016 Image-based modelling of nutrient movement in and around the rhizosphere. *J. Exp. Bot.* 67, 1059–1070. (doi:10.1093/jxb/erv544) 26739861 10.1093/jxb/erv544 PMC4753851

[22] Blunt MJ, Bijeljic B, Dong H, Gharbi O, Iglauer S, Mostaghimi P, Paluszny A, Pentland C 2013 Pore-scale imaging and modelling. *Adv. Water Resour.* 51, 197–216. (doi:10.1016/j.advwatres.2012.03.003)

[23] Roose T, Keyes SD, Daly KR, Carminati A, Otten W, Vetterlein D, Peth S 2016 Challenges in imaging and predictive modeling of rhizosphere processes. *Plant Soil* 407, 9–38. (doi:10.1007/s11104-016-2872-7)

[24] Schindelin J *et al.* 2012 Fiji: an open-source platform for biological-image analysis. *Nat. Methods* 9, 676–682. (doi:10.1038/nmeth.2019) 22743772 10.1038/nmeth.2019 PMC3855844

[25] Nye PH, Tinker PB (eds). 2000 *Solute movement in the rhizosphere*. Oxford, UK: Oxford University Press.

[26] Press WH, Teukolsky SA, Vetterling WT, Flannery BP 2007 *Numerical recipes: the art of scientific computing*. Cambridge, UK: Cambridge University Press.

[27] Drugan W, Willis J 1996 A micromechanics-based nonlocal constitutive equation and estimates of representative volume element size for elastic composites. *J. Mech. Phys. Solids* 44, 497–524. (doi:10.1016/0022-5096(96)00007-5)

[28] Schaap MG, Porter ML, Christensen BSB, Wildenschild D 2007 Comparison of pressure-saturation characteristics derived from computed tomography and lattice Boltzmann simulations. *Water Resour. Res.* 43, W12S06 (doi:10.1029/2006WR005730)

[29] Pot V *et al.* 2015 Three-dimensional distribution of water and air in soil pores: comparison of two-phase two-relaxation-times lattice-Boltzmann and morphological model outputs with synchrotron X-ray computed tomography data. *Adv. Water Resour.* 84, 87–102. (doi:10.1016/j.advwatres.2015.08.006)

[30] Peth S, Nellesen J, Fischer G, Horn R 2010 Non-invasive 3D analysis of local soil deformation under mechanical and hydraulic stresses by *μ*CT and digital image correlation. *Soil. Tillage. Res.* 111, 3–18. (doi:10.1016/j.still.2010.02.007)

[31] Carminati A 2013 Rhizosphere wettability decreases with root age: a problem or a strategy to increase water uptake of young roots? *Front. Plant. Sci.* 4, 1–9. (doi:10.3389/fpls.2013.00298) 23967001 10.3389/fpls.2013.00298 PMC3742997

[32] Cooper LJ, Daly KR, Hallett PD, Naveed M, Koebernick N, Bengough AG, George TS, Roose T 2017 Data from: Fluid flow in porous media using image-based modelling to parametrize Richards' equation University of Southampton Repository. (doi:10.5258/SOTON/405744) 10.1098/rspa.2017.0178 PMC5719621 29225490

