# Supplementary material for: Fluid flow in porous media using image-based modelling to parametrize Richards' equation
Source: Proc Math Phys Eng Sci. 2017 Nov 22;473(2207):20170178. doi: 10.1098/rspa.2017.0178 (PMC5719621; doi:10.1098/rspa.2017.0178)
Supplement: Supplementary Text [file rspa20170178supp1.docx]

**Fluid flow in porous media using image based modelling to parametrise Richards' equation**

**Supplementary Text**

**Experimental Verification of Modelled Results**

Water retention characteristics were obtained for the same soil and similar experimental conditions used for imaging. A standard Haines funnel setup was adapted to provide very high resolution information on wetting and drying characteristics by using a Zwick Z005 materials testing machine (Zwick GmbH & Co. KG, Ulm, Germany). A 5 N load cell, accurate to 2 mN, was placed at the base of the mechanical test frame and a 60 mm diameter No. 3 sintered glass funnel was attached to the moving crosshead with a displacement accuracy of 1 𝜇m. The sintered funnel was attached to a water reservoir that was on top of the load cell via a tube and fixed glass pipe. The tube went through a small hole at the top of the water reservoir, taking care to avoid contact with the sides so that the mass change of water could be recorded. This setup allowed for very accurate movement of the sintered funnel in relation to the surface of the water reservoir through a cycle of wetting and drying.

At the beginning of sample preparation the base of the sintered funnel was placed at -1000 Pa. A mass of 50 g of air-dried sieved soil was poured onto the funnel and allow to wet for 24 hours through capillarity to minimise slaking that may results from rapid saturation. The soil was then packed gently with a uniaxial compressive stress of 10 kPa, resulting in a dry bulk density of 1200 kg m^-3^. The sample was then saturated until the water volume entering the soil stabilised, followed by wetting and drying to minimum capillary pressure of -2500 Pa by raising and lowering the glass sintered funnel at a speed of 25 mm/hour. The mass of water in the liquid reservoir was recorded every 10 seconds, equivalent to a reading every 69 𝜇m.

From the volume of soil and its porosity, the saturation was determined. We used a particle density of 2.65 g cm^-3^ to convert from bulk density to porosity.
