# Supplementary material for: Fluid flow in porous media using image-based modelling to parametrize Richards' equation
Source: Proc Math Phys Eng Sci. 2017 Nov 22;473(2207):20170178. doi: 10.1098/rspa.2017.0178 (PMC5719621; doi:10.1098/rspa.2017.0178)
Supplement: Supplementary Figure 1 [file rspa20170178supp2.pdf]

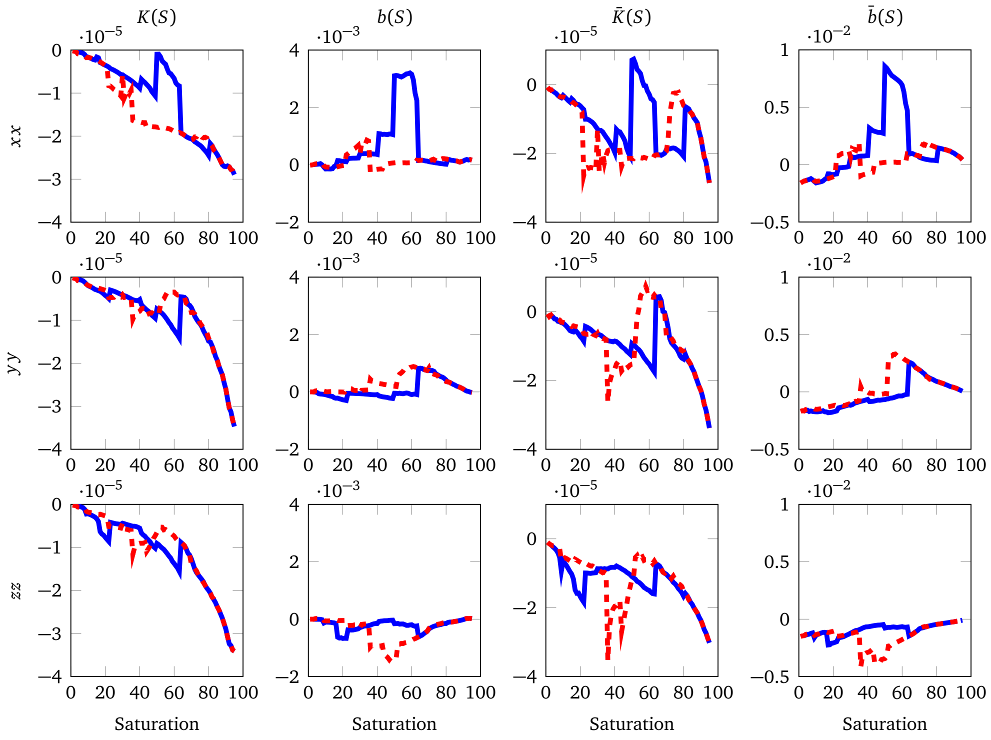

(a) Contact angle 20°

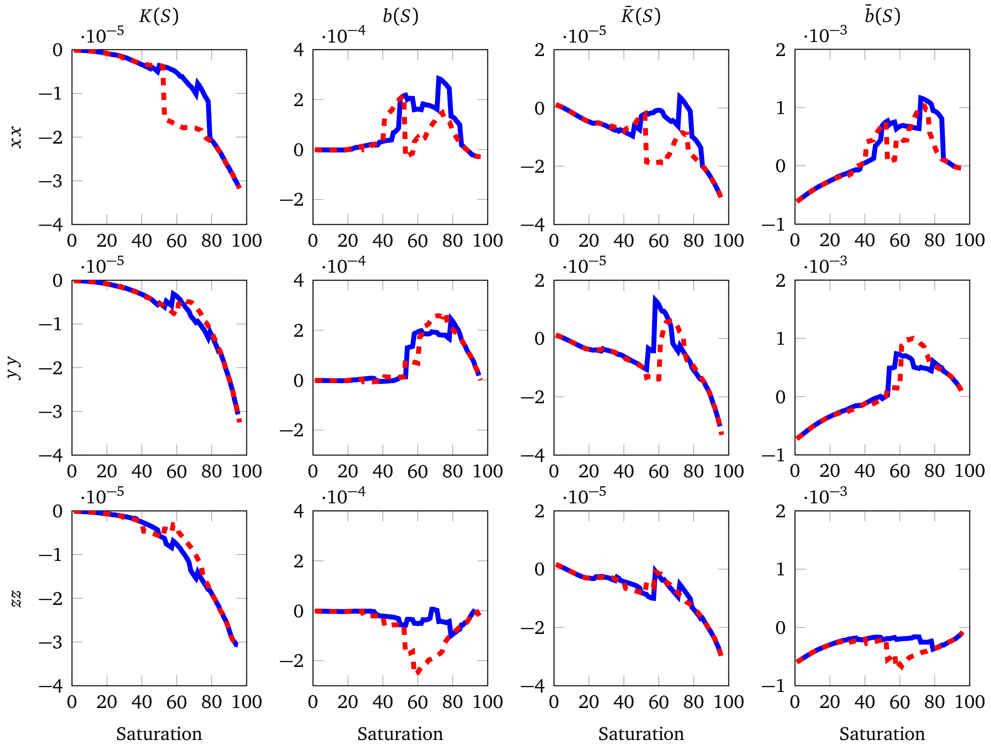

(b) Contact angle 0°

Figure 1: The components of $K(S)$, $b(S)$, $\bar{K}(S)$ and $\bar{b}(S)$ defined in equations (2.4). S is the relative water saturation of the pore space, $K(S)$ and $b(S)$ are the average velocities of the water for a unit drop in capillary and combined pressure, respectively. $\bar{K}(S)$ and $\bar{b}(S)$ are the average velocities of both the water and the air for a unit drop in capillary and combined pressure, respectively.
